# Demographic history and divergence of sibling grouse species inferred from whole genome sequencing reveal past effects of climate change

**DOI:** 10.1186/s12862-021-01921-7

**Published:** 2021-10-24

**Authors:** Kai Song, Bin Gao, Peter Halvarsson, Yun Fang, Siegfried Klaus, Ying-Xin Jiang, Jon E. Swenson, Yue-Hua Sun, Jacob Höglund

**Affiliations:** 1 Animal Ecology, Department of Ecology and Genetics, Uppsala University, Norbyvägen 18D, 75236 Uppsala, Sweden; 2 Key Laboratory of Animal Ecology and Conservation Biology, Institute of Zoology, Chinese Academy of Sciences, 100101 Beijing, People’s Republic of China; 3 Unit of Parasitology, Department of Biomedicine and Veterinary Public Health, Swedish University of Agricultural Sciences, PO Box 7036, 75007 Uppsala, Sweden; 4 Jena, Germany; 5 Faculty of Environmental Sciences and Natural Resource Management, Norwegian University of Life Sciences, PO Box 5003, 1432 Ås, Norway

**Keywords:** Boreal forest, Demographic history, Genomics, Ice age, Climate change, Qinghai–Tibetan plateau

## Abstract

**Background:**

The boreal forest is one of the largest biomes on earth, supporting thousands of species. The global climate fluctuations in the Quaternary, especially the ice ages, had a significant influence on the distribution of boreal forest, as well as the divergence and evolution of species inhabiting this biome. To understand the possible effects of on-going and future climate change it would be useful to reconstruct past population size changes and relate such to climatic events in the past. We sequenced the genomes of 32 individuals from two forest inhabiting bird species, Hazel Grouse (*Tetrastes bonasia*) and Chinese Grouse (*T*. *sewerzowi*) and three representatives of two outgroup species from Europe and China.

**Results:**

We estimated the divergence time of Chinese Grouse and Hazel Grouse to 1.76 (0.46–3.37) MYA. The demographic history of different populations in these two sibling species was reconstructed, and showed that peaks and bottlenecks of effective population size occurred at different times for the two species. The northern Qilian population of Chinese Grouse became separated from the rest of the species residing in the south approximately 250,000 years ago and have since then showed consistently lower effective population size than the southern population. The Chinese Hazel Grouse population had a higher effective population size at the peak of the Last Glacial Period (approx. 300,000 years ago) than the European population. Both species have decreased recently and now have low effective population sizes.

**Conclusions:**

Combined with the uplift history and reconstructed climate change during the Quaternary, our results support that cold-adapted grouse species diverged in response to changes in the distribution of palaeo-boreal forest and the formation of the Loess Plateau. The combined effects of climate change and an increased human pressure impose major threats to the survival and conservation of both species.

**Supplementary Information:**

The online version contains supplementary material available at 10.1186/s12862-021-01921-7.

## Background

To understand the possible effects of on-going and future climate change it would be useful to reconstruct past population size changes and relate such to climatic events in the past. Analyses of whole genome sequences can be used to infer past demographic events and has been used in the past to infer effects of major climatic events in the evolutionary past of study organisms [1–3]. Here we use whole genome sequences obtained from individuals belonging to a pair of boreal forest dwelling sibling grouse species: the Chinese Grouse *Tetrastes sewerzowi* and Hazel Grouse *T*. *bonasia* to infer their demographic histories and relate the inferred changes to known past climatic geological events and their climatic impacts [4]. The demographic history of a species can be examined through reconstruction of the effective population size, using the information embedded in the genome sequences of a diploid species [1].

The boreal forest, one of the largest biomes on Earth, is inhabited by a large number of species [5–7]. Most of the boreal forest is found throughout the high northern latitudes from about 50°N to 70°N, but boreal forest also occurs in mountain regions further to the south, such as at the southeastern edge of the Qinghai–Tibet Plateau (QTP). These forest areas used to be connected to the more widely distributed boreal forest to the north, but retracted to present distributions during the uplifting of the plateau [8]. The global climate change and uplift of QTP had significant influence on divergence and demographic history of many sibling faunas in Eurasia and the QTP boreal forest. Hazel Grouse and Chinese Grouse, the sibling species of concern in this study show adaptions to cold environments, for example by having feathered legs and nostrils [9–11]. The origin of the cold-adapted Pleistocene species, especially megafauna, has usually been sought either in the arctic tundra or in the cool steppes outside the QTP [12, 13]. However, an alternative scenario, called the “out of Tibet hypothesis”, has been offered, based on new fossil assemblages [14–16]. Here it is argued that the evolution of present-day animals in the Arctic region is intimately connected to ancestors that first became adapted for life in cold regions in the high-altitude environments of the QTP (2.6 to 5 Mya), and were thus pre-adapted to cold climates during the ice age (2.6 to 0.1 Mya) [14–16].

During the Quaternary, vast areas of the boreal forest were repeatedly affected by major glaciations. Ice sheets that formed over Scandinavia spread eastwards across the northwestern Russian plains and southwards across northern and middle Siberia [17]. However, the ice sheets did not cover eastern Siberia. This led to a fauna in eastern Siberia that is considerably older and richer, with more endemic species and genera from various taxa, than that of the western Siberian boreal forest [17–22]. The historical glacial-interglacial cycles dramatically influenced the fluctuations and long-term declines in effective population size for some species [2, 23–25]. Therefore, the glaciations had a significant influence on the present genetic structure of populations, species, and communities [22, 26, 27].

Before the Quaternary Period, the global average temperature in the mid-Pliocene (3.3–3 Mya) was 2–3 ℃ higher than today, whereas carbon dioxide levels were the same [28]. The change to a cooler, drier, and more seasonal climate had considerable impacts on the Pliocene vegetation, reducing tropical species worldwide, and coniferous forests and tundra covered much of the north [29]. During the Late Miocene and the Pliocene periods, the QTP had an expanding forest cover (up to between 1000 and 2000 m) [30–33], particularly at its eastern edge, currently located in the Chinese provinces of Qinghai, Sichuan, and Yunnan. During the Quaternary period, the boreal forest experienced many climate-induced fluctuations [7]. Several glacial and interglacial periods, along with the uplift of the QTP and accumulation of the Loess Plateau, promoted the geographic distribution pattern of boreal forests seen today.

During the Pleistocene-Holocene (1.10–0.60 Ma), the QTP experienced three rapid uplifting stages when mountain ranges formed. The climate changed from wet and warm to dry and cold, with cryosphere (year round ice) on the plateau and forest receding to the edge. About 0.15 Ma, the climate became colder and drier, and 10 kya the forests extended even higher [34, 35]. The rise of the QTP and increase in global ice volume had a great influence on the interior Asian aridification, especially for the formation of the Loess Plateau from sediment deposited by wind-blown dust since 2.40 Ma [36–39]. In the early Quaternary, the warm and moist forest ecological environment found in the Tertiary continued on the Loess Plateau. During the middle and late Pleistocene, the climate gradually became drier and the vegetation changed to an arid grassland. These patterns are not only consistent with the changing characteristics of the fossil assemblage, but are also consistent with the formation of the loess area [40–42].

The Chinese Grouse is an endemic species inhabiting high mountain coniferous forests in central China [4, 43]. The Hazel Grouse is the sibling species of Chinese Grouse and occurs within the temperate, boreal, and subarctic biogeographical zones of the Northern Hemisphere [44, 45]. Studies on speciation of the sibling species show conflicting patterns [9, 46, 47], but previous work support a divergence in the mid-Pleistocene and the beginning of the Quaternary. Here we used whole genome resequencing data to estimate the divergence time and used a pairwise sequentially Markovian coalescent (PSMC) modeling to reconstruct the ancestral demographic trends in both species. Finally, we used a multiple sequentially Markovian coalescent (MSMC) model to estimate the effective population size of both species in more recent time.

## Results

### Phylogenetic relationships

**Fig. 1:**
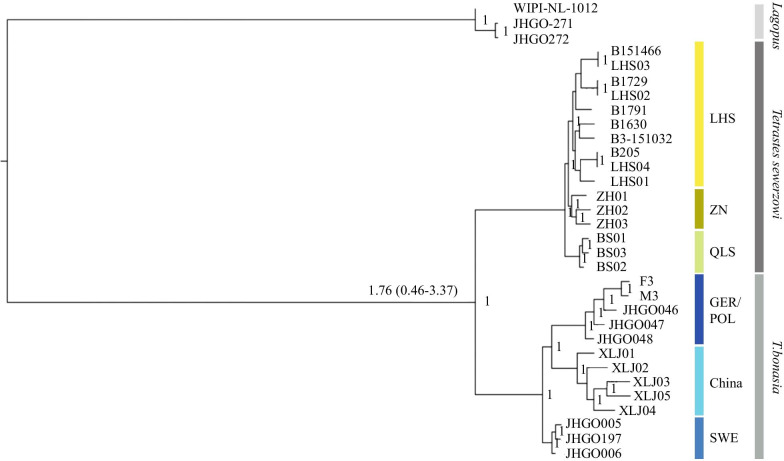
Phylogenetic relationships based on Bayesian analyses. The split between these sibling species is given in million years (with 95% confidence limits) from MSMC analysis. Numbers at the nodes indicate posterior probabilities

The trees based on the three different methods (Bayesian, Maximum Likelihood and Neighbor Joining) used to infer the phylogenetic relationships largely concur and only show minor differences among the different populations of Hazel Grouse (Fig. 1, Additional file 1: Figs. S1, S2). In the Bayesian and ML analyses, the China and GER/POL populations are sister to each other and in the NJ analyses the GER/POL and SWE populations are sister to each other. All analyses suggest clear species and population structure. Using the Bayesian tree, the split between the Chinese Grouse and Hazel Grouse was estimated to 1.76 (0.46–3.37) MYA. In all analyses the three populations of Chinese Grouse were clearly separated and the branching patterns was the same among populations. The Swedish population appeared to be clearly separated from the populations in Germany and Poland. Thus the broad phylogenetic relationships among the sampled species were independent of phylogenetic reconstruction method, as maximum-likelihood and Neighbor joining methods yielded the same topologies (Fig. 1, Additional file 1: Fig. S1, S2).

### Population demographic histories

**Fig. 2:**
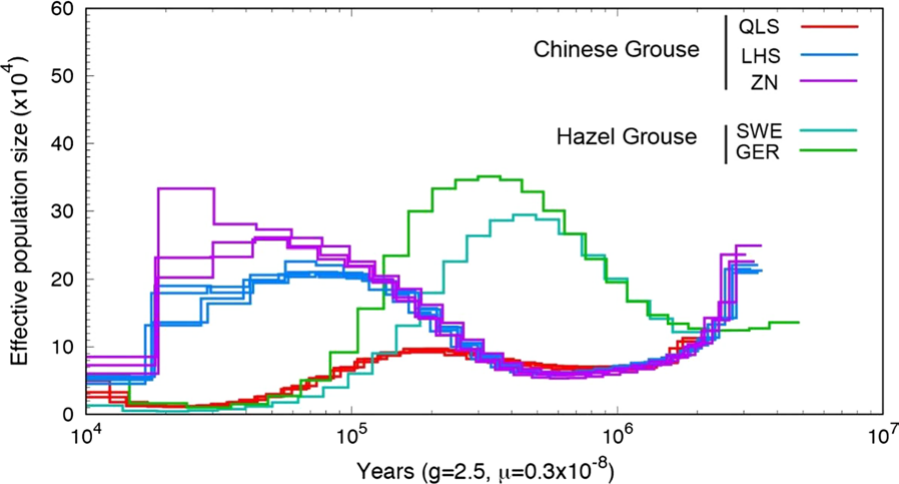
Historical changes in effective population size of Chinese Grouse and Hazel Grouse from PSMC analyses of whole-genome sequences. Profiles labeled SWE and GER, the Europan populations, (green) are from Hazel Grouse, ZN (olive), LHS (blue) and QLS (red) are from Chinese Grouse

**Fig. 3:**
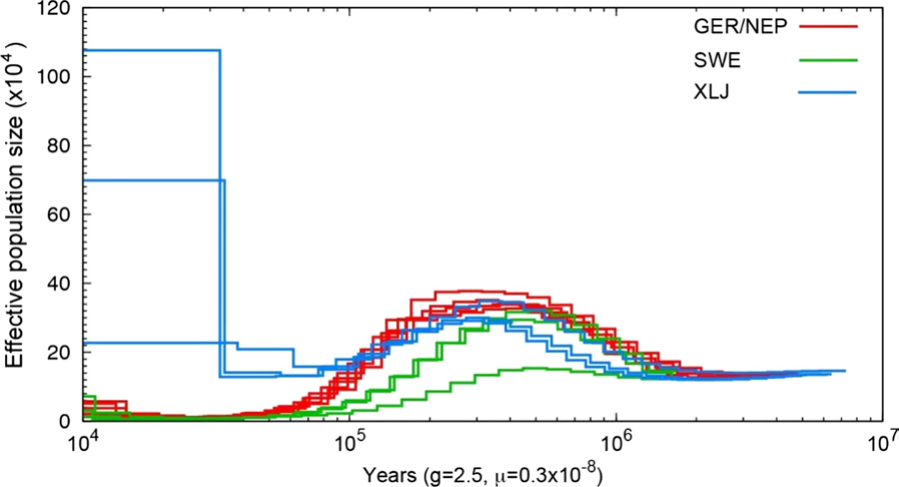
Historical changes of effective population size in Hazel Grouse from PSMC analyses of whole-genome sequences. Profiles SWE (green) are from Jämtland, Sweden, XLJ (blue) are from China and GER/NEP (red) are of German/Polish ancestry

An analysis of full diploid genomes from the sibling species using PSMC modelling clearly showed different results for the two species (Fig. 2). For the Chinese Grouse, the southern (LHS and ZN) and northern populations (QLS) had different demographic histories. The northern population experienced persistently low effective population sizes and has declined since about 0.2 Mya. The demography of the southern Chinese Grouse population showed a drastic decline around 17,000 years ago and population peak at 20–30,000 years ago (Fig. 2). For the Hazel Grouse, the population peaked around 120,000 years ago (Fig. 2). Since 100,000 years ago the population trends differed between the Chinese and European Hazel Grouse populations (Fig. 3). All the European populations appeared to have undergone persistent declines in population size after the peak 0.3 Mya. In contrast, population size estimates obtained from the Chinese Hazel Grouse samples showed an increase to a large effective population size during the Last Glacial Maximum (LGM) 20,000 years ago. Since then, Hazel Grouse in China showed consistently higher population sizes than European Hazel Grouse (Fig. 3).

**Fig. 4:**
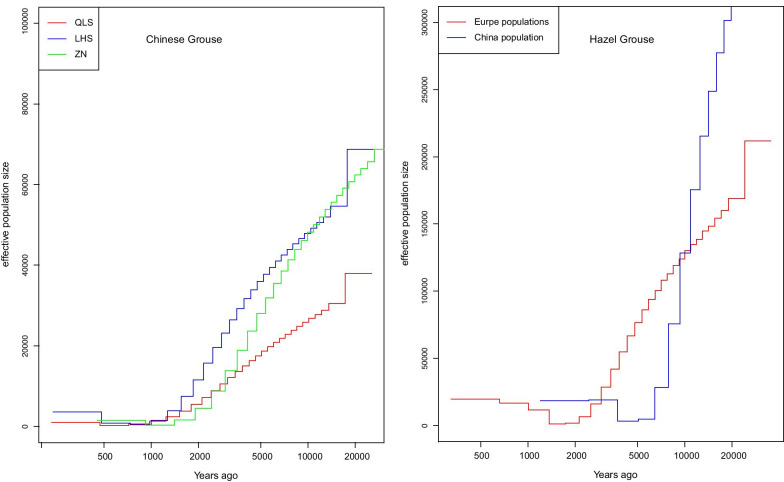
Effective population size of Chinese Grouse (left) and Hazel Grouse (right) inferred using Multiple Sequentially Markovian coalescent (MSMC) models

The MSMC analysis of the demographic history of the two grouse species from ~ 20,000 years ago to the present suggested that population sizes have decreased constantly since then to 2000 years ago with a decline in Chinese Hazel Grouse starting from higher levels than the European populations (Fig. 4).

## Discussion

We have performed the first population-scale, whole genome sequencing studies of the sibling species Chinese Grouse and Hazel Grouse, inhabiting the QTP and Eurasian boreal forests, respectively. The analyses presented here provide insights into the phylogenetic relationships, ancestral population demography and recent time population decreases in relation to changes in climate and related factors in different populations of these two species. Our PSMC analyses covered a period from 1 Mya to 20,000 years ago (Fig. 2), a period covering the chronological distribution of four grouse genera (*Tetrao, Bonasa, Lagopus and Dendragapus*) [44].

Our whole genome data allowed us to estimate relationships between Chinese Grouse and Hazel Grouse and among different populations of the two sibling species. We estimated the divergence time of the two *Tetrastes* species to 1.7 (0.46–3.37) Mya using whole genome data, whereas a previous study estimated the same divergence to approximately 0.8–2.5 Mya based on ultra-conserved element sequences [9]. In the Pliocene period (5.33–2.58 Mya), the climate was similar to the present [28]: a cool, dry, seasonal climate, which had considerable impacts on the vegetation. All grouse species occur within the temperate, boreal, and Arctic biogeographical zones of the Northern Hemisphere and are adapted to cold climates [44]. The extinct *Bonasa praebonasia* is ancestral to the sibling species Chinese Grouse and Hazel Grouse [4, 43, 46]. Prior to the Quaternary period it is assumed that, *B. praebonasia* populations spread throughout the existing coniferous forests and crossed into the QTP. In the beginning of the Quaternary, with cyclic growth and decay of continental ice sheets [48, 49], the distribution patterns of boreal forests and steppes changed worldwide. The uplifting of the QTP and the continuous accumulation of loess in the Loess Plateau was the main driving factor to limit the distribution of boreal forest in China and divide the ancestral species in to sibling species.

The conifer-dominated forests with deciduous trees on the southeastern edge of QTP, in which only the wetter northern slopes have forest vegetation, resulted in fragmentation of the habitat for Chinese Grouse [43]. It has been shown that large-scale deforestation, intensive livestock grazing, and climate change exacerbates habitat loss and fragmentation [50–53]. Similar to other mountainous species [54, 55], the distribution of Chinese Grouse would likely have shifted in elevation as the coniferous forest changed under different climate change scenarios [3, 52]. However, unlike species that have greater dispersal capabilities, the Chinese Grouse has had limited to no contact with Hazel Grouse [56] and the treeless Loess Plateau has acted as a barrier to gene flow among the Northern Taiga and the Northeastern QTP.

The sequenced QLS birds were from the Qilian Mountains, which are situated on the northeastern edge of the QTP; the coniferous forests there are isolated from the southern QTP. Several rare and endemic gallinaceous bird species are found in the Qilian Mountains [57, 58]. Our results show that the northern population of Chinese Grouse (QLS) has a demographic history different from those in the south, probably as a consequence of isolation and small effective population sizes throughout their history. This patterns is especially visible during the Last Glacial Period (LGP, 115,000–11,700 years ago), where the pattern between the northern QLS population and the two southern populations (LHS and ZN) are different (Figs. 1 and 2). Based on our data, these populations have been split for a long time. Thus, gene flow among the southern populations and northern population is extremely unlikely.

The Qilian Mountains has served as a refuge, not only for the Chinese Grouse, but also for other gallinaceaous birds during the Pleistocene glaciations, such as the Blood Pheasant (*Ithaginis cruentus*), Chukar Partridge (*Alectoris chukar*), and Tibetan Partridge (*Perdix hodgsoniae*). These forest specialist gallinaceaous birds gradually withdrew from large parts of northern China as a consequence of the plateau uplift, the formation of the Loess Plateau, and loss of forest in these areas. Populations remained in isolated refugia (e.g. QLS) during the glaciations that occurred during the Quaternary glacial period [59]. In contrast to the Chinese Grouse from the Qilian Mountains, the two southern populations have similar PSMC profiles and are more closely related (Fig. 2). The effective population size in these two populations showed a peak during the LGM and then decreased. This supports the notion that the LGM had a less dramatic effect on these populations than on forest species in Europe and North America and is a distinct regional feature [60].

**Fig. 5:**
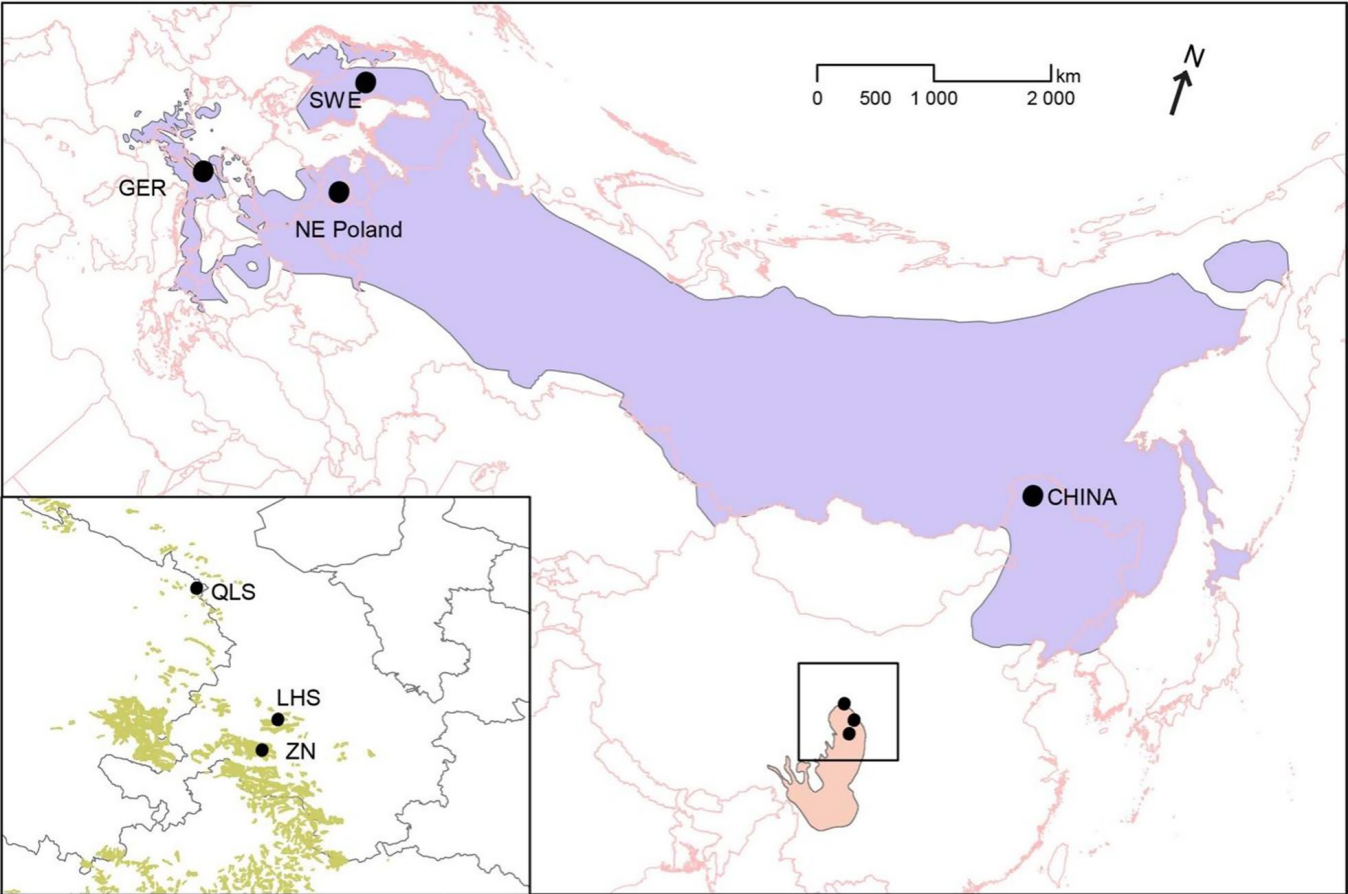
Distribution and sampling localities of the Chinese Grouse (red) and the Hazel Grouse (blue). The fine scale distribution of Chinese Grouse (green) is inset in the lower left corner. The distribution area was downloaded from “*The IUCN Red List of Threatened Species*, which is not protected by copyright, https://www.iucnredlist.org” (IUCN 2020)

The Hazel Grouse inhabits dense coniferous forest, preferably with spruces (*Abies*) mixed with deciduous trees like *Alnus*, *Betula*, and *Salix*, and has limited dispersal capability and narrow habitat requirements [4]. It is a sedentary species and its distribution range is continuous across northern Eurasia from Hokkaido in the east to Western Europe in the west. It is classified as Lower Risk (least concern) by the IUCN [53], but red-listed in some central and southern European countries [4]. Many remaining Hazel Grouse populations are scattered and small, because of forest fragmentation and limited dispersal [61]. Our population sampling of this species includes samples from China, Sweden, and central Europe (Fig. 5). Our results suggest that the Chinese population is genetically distinct from the Swedish and the Central European populations, probably as a result of isolation by distance and accelerated genetic drift in smaller and more isolated populations in Europe [62].

The Pleistocene environment and vegetation in Eurasia are important factors to take into account when discussing molecular divergence among Hazel Grouse [27, 63]. The PSMC results show that population size of European Hazel Grouse populations decreased since 120,000 years ago and the population was constantly at low numbers until 12,000 years ago, a time period which also coincides with the LGP. During the LGP, the ice sheet stretched from the northern parts of the British Isles, Germany, Poland, and the Taymyr Peninsula in western Siberia, with the deepest ice sheet over Scandinavia [64]. In contrast, northeastern Siberia was not covered by a continental-scale ice sheet [65] and may have served as a refugium for Hazel Grouse and other forest species. Thus, Chinese populations of Hazel Grouse remained relatively large during the LGP. Our results on Hazel Grouse are supported by studies showing that the fauna in the eastern Siberian boreal taiga is older and richer than that in western Siberia [18–21].

Our MSMC results suggest that after the LGP, N_e_ decreased dramatically from 10,000 years ago to the present for both sibling species. Additionally, the MSMC analysis of the sibling grouse species from ~ 20,000 years ago to the present suggested that population sizes have decreased constantly from 20,000 years ago to 200 years ago. The dramatic decreases in N_e_ from 10,000 years ago to the present, coincide with the climate change in the Holocene (10,000 years ago to 5000 years ago) and increasing human forest exploitation from 8500 years ago [66]. In the early Holocene, around 10,000 years ago, the interglacial period was firmly established, exhibiting a warmer and moister climate than today. The ice cover regressed and trees were able to re-colonize the northern taiga. However, at the beginning of the Neolithic Revolution (8500 years ago), humans started to exploit forests, not only for wood and food, but also for space [67, 68]. Nowadays, boreal ecosystems world-wide are threatened by direct human activity and climate change [7].

## Conclusions

In conclusion, our analyses provided insights into divergence and demographic history of a sibling species pair both residing in cold boreal forest habitats in Eurasia and the QTP. Combined with the uplift history and reconstructed climate change during the Quaternary, our results support that cold-adapted grouse species diverged in response to changes in the distribution of palaeo-boreal forest and the formation of the Loess Plateau. Our analyses also provide insights in to how these sibling species have responded to changes in climate throughout their evolutionary history. In a recent study [3] we were able to show how the changes in population size of Chinese Grouse were related to climate induced shifts in the distribution of available habitat. Combined with the results of this study, they provide evidence of population resilience in the face of dramatic climatic fluctuations. However, smaller and fragmented populations are facing higher risks of loss of genetic diversity and extinction as exemplified by the Qilian population of Chinese Grouse. Our results also hint at anthropogenic induced stress in recent times, and thus unrelated to major climatic shifts, has reinforced the population declines of both species. The combined effects of climate change an increased human pressure thus impose major threats to the survival and conservation of both species.

## Methods

### Sampling and data collection

A total of 29 samples from the two sibling grouse species were collected from 8 locations; 16 Chinese Grouse samples from three populations from Gansu, China (3 from Beishan in the Qilian Mountains (QLS); 3 from Zhuoni County (ZN); 10 from the Lihanhuashan National Nature Reserve (LHS)) along with 13 Hazel Grouse samples from five populations (1 from Northeastern Poland (NEP); 1 from the Austrian Alps; 3 from the Bavarian forest, Germany (GER); 3 from Jämtland, Sweden (SWE); and 5 from Northeastern China (XLJ)) (Fig. 5, Additional file 1: Table S1). All the Chinese Grouse samples and the blood samples of Hazel Grouse from the XLJ population were collected in the field. The Hazel Grouse samples labeled F3 (with ancestry in NE Poland) and MR (with ancestry from the Austrian Alps) came from a captive stock at a breeding station in Germany. All other Hazel Grouse were obtained as muscle tissue from hunted individuals. We also used 3 *Lagopus* samples as an outgroup (2 Rock Ptarmigan *L. muta* and 1 Willow Grouse *L*. *lagopus*). All samples were collected and preserved in 99% ethanol and stored at −20 °C.

### Resequenced genomes

All samples were sequenced using the Illumina sequencing platform (Illumina PE 150, Hi-Seq 4000) at *Annoroad Gene Technology* (Beijing, China). We used a Gentra Puregene Blood kit (Qiagen) to extract the genomic DNA from all samples according to the manufacturer’s (Illumina) Instructions. Then we assessed the quality of DNA by electrophoresis on 1% agarose gel and the quantity of DNA by a BioDrop mLITE spectrophotometer (a total of 15 mg of DNA was quantified using the spectrophotometer). Our analyses were based on cleaned reads, which were filtered following a three-step procedure: (1) removal adapter polluted reads > 5 bp, (2) removing low-quality reads with quality score < 19, and (3) sequence reads where Ns comprised > 5% were removed (additional file 2 Data filter summary and distribution). After filtering, a total of 686.04 Gb (97%, of 705.13 Gb) high-quality paired reads remained for further analysis.

Trimmomatic v0.33 was used to trim the Illumina fastq files and remove adapters, based on manufacturer’s adapter sequences. Raw data of fastq format were then processed with in-house perl scripts. In this step, clean data were obtained by removing reads containing adapter, reads containing poly-N, and low-quality reads from raw data. At the same time, Q20, Q30, and GC content of the clean data was calculated. All the downstream analyses were based on the clean data with high quality (Q30). The clean reads were mapped to the Chinese Grouse reference genome that we assembled [3] by BWA 0.7.5a [69], with parameters: aln -o 1 -e 10 -t 4 -l 32 -i 15 -q 10, and reads having a mean of approximately 15 × depth for each individual and > 90% coverage of the Chinese Grouse genome were retained for SNP calling. We employed a Bayesian algorithm in Samtools 0.1.19 [70] to call SNPs using the command ‘mpileup’ with parameters as ‘-q 1 -C 50 -S -D -m 2 -F 0.002 -u’. We calculated the genotype likelihoods from reads for each individual at every genomic location and estimated the allele frequencies. We used GATK version 3.2–2 [71] to call variations including SNPs and indels. We filtered SNPs using VCFtools v0.1.11 [72] and by following the criteria: coverage depth ≥ 4 and ≤ 1000 (which nearly twice of the average sequencing depth 16 × 32 sample); root mean square (RMS) mapping quality ≥ 20; the distance of adjacent SNPs ≥ 5 bp (this will exclude potential misaligned nucleotides); the distance to a gap ≥ 5 bp; reads mapping quality value ≥ 30 (this will exclude the reads misallocated to different site over the genome). SNPs with minor allele frequency (MAF) value under 0.05 were excluded with vcftools -maf 0.05 (this help us to exclude the SNPs of low frequency in the population for the common procedure of population genetics research). Haplotype missing call rate ≥ 0.05 were also excluded by in-house perl script (to get high call rate of SNPs among each individuals).

### Phylogenetic trees construction

To estimate phylogenetic relationships, all SNPs were used to calculate the pairwise genetic distances among all samples. A neighbor-joining (NJ) tree with 100 bootstrap replicates was inferred using TreeBeST 1.9.2 [73] using all samples. Whole genome sequences from one Willow Ptarmigan from Newfoundland, Canada, and two Rock Ptarmigan from western Greenland were included as an outgroup. RAxML version 8.2.10 was used to infer phylogenetic trees with the maximum likelihood (ML) method based on the same dataset. MrBayes 3.2.7a × 86_64 was used to perform Bayesian inference of phylogeny with the parameters: lset nst = 6 rates = gamma, Ngen = 800,000 Samplefreq = 10 Printfreq = 100, sump burninfrac = 0.2, sumt burninfrac = 0.2. A MrBayes consensus tree was extracted with posterior probability supporting rates. MCMCtree contained in the PAML software provided Bayesian methods to estimate divergence times of genomic-sized sequences between populations and species [74]. When the average standard deviation of split frequencies went under 0.01 which means convergence, the burnin was set to (ngen/samplefreq) * 0.25 and then sumt was used to get the convergence tree.

### Demographic history inferred from PSMC and MSMC

Demographic history reconstruction using a PSMC model can estimate changes in effective population size between 20 kya and 3 Mya [1]. Because the power of PSMC is quite limited for predictions more recent than 20 kya, MSMC analyses can be performed to recover more recent trends in effective population using multiple genome sequences [1, 75]. The demographic history of the two grouse species was inferred using PSMC modelling, as described in Li and Durbin [1], and MSMC analyses [75]. To run PSMC and MSMC, we assumed two parameters: the generation time (2.5 years) [43] and the mutation rate per generation (0.3 × 10^–8^ per nucleotide per year) [76]. The mutation rate (per nucleotide per year, μ) was selected from studies of Willow Grouse (0.299 × 10^–8^) and Rock ptarmigan (0.310 × 10^–8^) [76, 77]. PSMC was run for 100 iterations using an initial h = q ratio of 5 and the default time patterning. Bootstrapping was performed according to Li and Durbin [1] by resampling 500,000 bp chunks of the genome with replacement to perform 100 bootstrap replicates.

## Supplementary Information

**Additional file 1: Table. S1.** Sample information and the statistic of whole genome quality control. **Fig. S1.** Estimated ancestral relationships of Grouse based on Maximum Likelihood using RAxML. **Fig. S2.** Neighbor-joining tree constructed from Nei’s standard genetic distances of the whole genome sequences of Chinese and Hazel grouse. Numbers at the nodes indicate bootstrap support. Substitution rate is indicated below the figure.

## Data Availability

Sequencing data for the Chinese Grouse and Hazel Grouse have been deposited in Short Read Archive under Project Number PRJNA588719 and PRJCA005913.

## Abbreviations

QTP: Qinghai–Tibetan Plateau
SDM: Species Distribution Modelling
PSMC: Pairwise Sequentially Markovian Coalescent
MSMC: Multiple sequentially Markovian coalescent
ML: Maximum likelihood
MID: Mid-Holocene
LGM: Last glacial maximum
LGP: Last glacial period
LIG: Last interglacial
