# Supplementary material for: Demographic history and divergence of sibling grouse species inferred from whole genome sequencing reveal past effects of climate change
Source: BMC Ecol Evol. 2021 Oct 24;21:194. doi: 10.1186/s12862-021-01921-7 (PMC8543876; doi:10.1186/s12862-021-01921-7)
Supplement: Supplementary file 1 — Additional file 1: Table. S1. Sample information and the statistic of whole genome quality control. Fig. S1. Estimated ancestral relationships of Grouse based on Maximum Likelihood using RAxML. Fig. S2. Neighbor-joining tree constructed from Nei’s standard genetic distances of the whole genome sequences of Chinese and Hazel grouse. Numbers at the nodes indicate bootstrap support. Substitution rate is indicated below the figure. [file 12862_2021_1921_MOESM1_ESM.docx]

Additional file 1

Table. S1. Sample information and the statistic of whole genome quality control.

| **Species** | **Location** | **Sample** | **Raw Reads** | **Raw Bases** | **Clean Reads** | **Clean Bases** | **Error Rate** | **Q20** | **Q30** | **GC Content** |
| --- | --- | --- | --- | --- | --- | --- | --- | --- | --- | --- |
| Chinese Grouse | LHS | LHS01 | 143 628 342 | 21.54G | 141 357 988 | 21.2G | 0,01% | 97,52% | 94,18% | 41,94% |
| Chinese Grouse | LHS | LHS02 | 139 214 378 | 20.88G | 137 702 462 | 20.66G | 0,01% | 97,86% | 94,86% | 41,61% |
| Chinese Grouse | LHS | LHS03 | 161 232 574 | 24.18G | 159 463 010 | 23.92G | 0,01% | 97,69% | 94,48% | 41,77% |
| Chinese Grouse | LHS | LHS04 | 123 347 872 | 18.5G | 121 834 992 | 18.28G | 0,01% | 97,71% | 94,58% | 41,74% |
| Chinese Grouse | LHS | B151466 | 152 899 516 | 22.93G | 148 601 096 | 22.29G | 0,02% | 96,96% | 92,91% | 41,68% |
| Chinese Grouse | LHS | B1630 | 138 806 838 | 20.82G | 133 756 888 | 20.06G | 0,02% | 97,01% | 93,12% | 42,69% |
| Chinese Grouse | LHS | B1729 | 142 167 828 | 21.32G | 138 484 602 | 20.77G | 0,02% | 97,08% | 93,19% | 41,16% |
| Chinese Grouse | LHS | B1791 | 158 510 302 | 23.77G | 151 902 920 | 22.79G | 0,02% | 96,98% | 93,12% | 43,19% |
| Chinese Grouse | LHS | B205 | 153 155 858 | 22.97G | 147 789 652 | 22.17G | 0,02% | 96,93% | 92,95% | 43,60% |
| Chinese Grouse | LHS | B3-151032 | 135 113 920 | 20.26G | 129 715 362 | 19.46G | 0,02% | 96,68% | 92,46% | 42,68% |
| Chinese Grouse | QLS | BS01 | 149 007 364 | 22.35G | 147 214 746 | 22.08G | 0,01% | 97,68% | 94,51% | 41,94% |
| Chinese Grouse | QLS | BS02 | 140 641 794 | 21.09G | 139 032 212 | 20.85G | 0,01% | 97,72% | 94,58% | 42,16% |
| Chinese Grouse | QLS | BS03 | 128 238 154 | 19.23G | 126 787 218 | 19.02G | 0,01% | 97,65% | 94,37% | 42,04% |
| Chinese Grouse | Zhuoni | ZN01 | 110 715 764 | 16.6G | 109 339 154 | 16.4G | 0,01% | 97,69% | 94,52% | 42,35% |
| Chinese Grouse | Zhuoni | ZN02 | 128 906 698 | 19.33G | 127 424 978 | 19.11G | 0,01% | 97,81% | 94,77% | 41,89% |
| Chinese Grouse | Zhuoni | ZN03 | 146 153 638 | 21.92G | 143 980 604 | 21.6G | 0,01% | 97,61% | 94,35% | 42,31% |
| Hazel Grouse | China | XLJ01 | 127 430 906 | 19.11G | 124 639 170 | 18.7G | 0,01% | 97,25% | 93,88% | 42,62% |
| Hazel Grouse | China | XLJ02 | 106 533 868 | 15.98G | 104 792 878 | 15.72G | 0,01% | 97,60% | 94,36% | 42,35% |
| Hazel Grouse | China | XLJ03 | 127 053 362 | 19.05G | 124 289 282 | 18.64G | 0,01% | 97,63% | 94,62% | 43,00% |
| Hazel Grouse | China | XLJ04 | 149 054 212 | 22.35G | 146 683 052 | 22G | 0,01% | 97,72% | 94,52% | 42,23% |
| Hazel Grouse | China | XLJ05 | 177 222 072 | 26.58G | 174 237 468 | 26.14G | 0,01% | 97,46% | 94,22% | 42,49% |
| Hazel Grouse | Sweden | JHGO005 | 141 679 556 | 21.25G | 138 673 432 | 20.8G | 0,01% | 97,00% | 93,31% | 43,46% |
| Hazel Grouse | Sweden | JHGO006 | 138 957 440 | 20.84G | 111 051 668 | 16.66G | 0,01% | 97,36% | 93,95% | 49,15% |
| Hazel Grouse | Sweden | JHGO197 | 136 884 104 | 20.53G | 134 306 646 | 20.15G | 0,01% | 97,43% | 94,01% | 42,53% |
| Hazel Grouse | Germany | JHGO046 | 142 837 302 | 21.42G | 139 529 196 | 20.93G | 0,01% | 97,38% | 94,08% | 42,77% |
| Hazel Grouse | Germany | JHGO047 | 138 790 648 | 20.81G | 136 819 280 | 20.52G | 0,01% | 97,63% | 94,39% | 42,05% |
| Hazel Grouse | Germany | JHGO048 | 165 487 810 | 24.82G | 162 663 952 | 24.4G | 0,01% | 97,37% | 93,91% | 42,58% |
| Hazel Grouse | Germany | F3 | 174 722 516 | 26.2G | 170 426 708 | 25.56G | 0,02% | 96,60% | 92,21% | 43,84% |
| Hazel Grouse | Germany | M3 | 147 096 620 | 22.06G | 142 298 546 | 21.34G | 0,02% | 96,62% | 92,27% | 42,35% |
| Rock Ptarmigan | Newfoundland | WIPI-NL-1012 | 162 187 862 | 24.32G | 157 268 684 | 23.59G | 0,02% | 96,95% | 92,99% | 43,20% |
| Rock Ptarmigan | Greenland, DK | JHGO-271 | 165 026 116 | 24.75G | 158 321 760 | 23.75G | 0,01% | 97,05% | 93,23% | 42,39% |
| Willow Ptarmigan | Greenland, DK | JHGO272 | 112 075 052 | 16.81G | 108 520 580 | 16.28G | 0,01% | 97,20% | 93,44% | 42,37% |


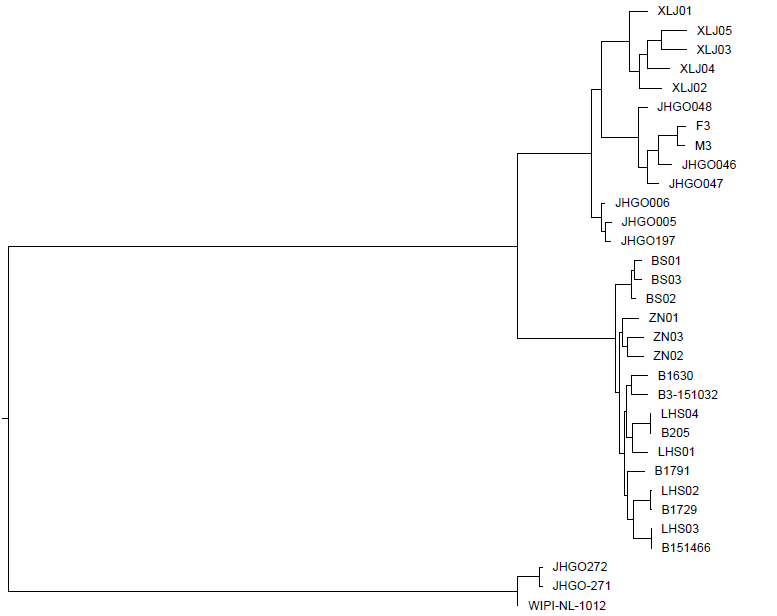


Fig. S1. Estimated ancestral relationships of Grouse based on Maximum Likelihood using RAxML.


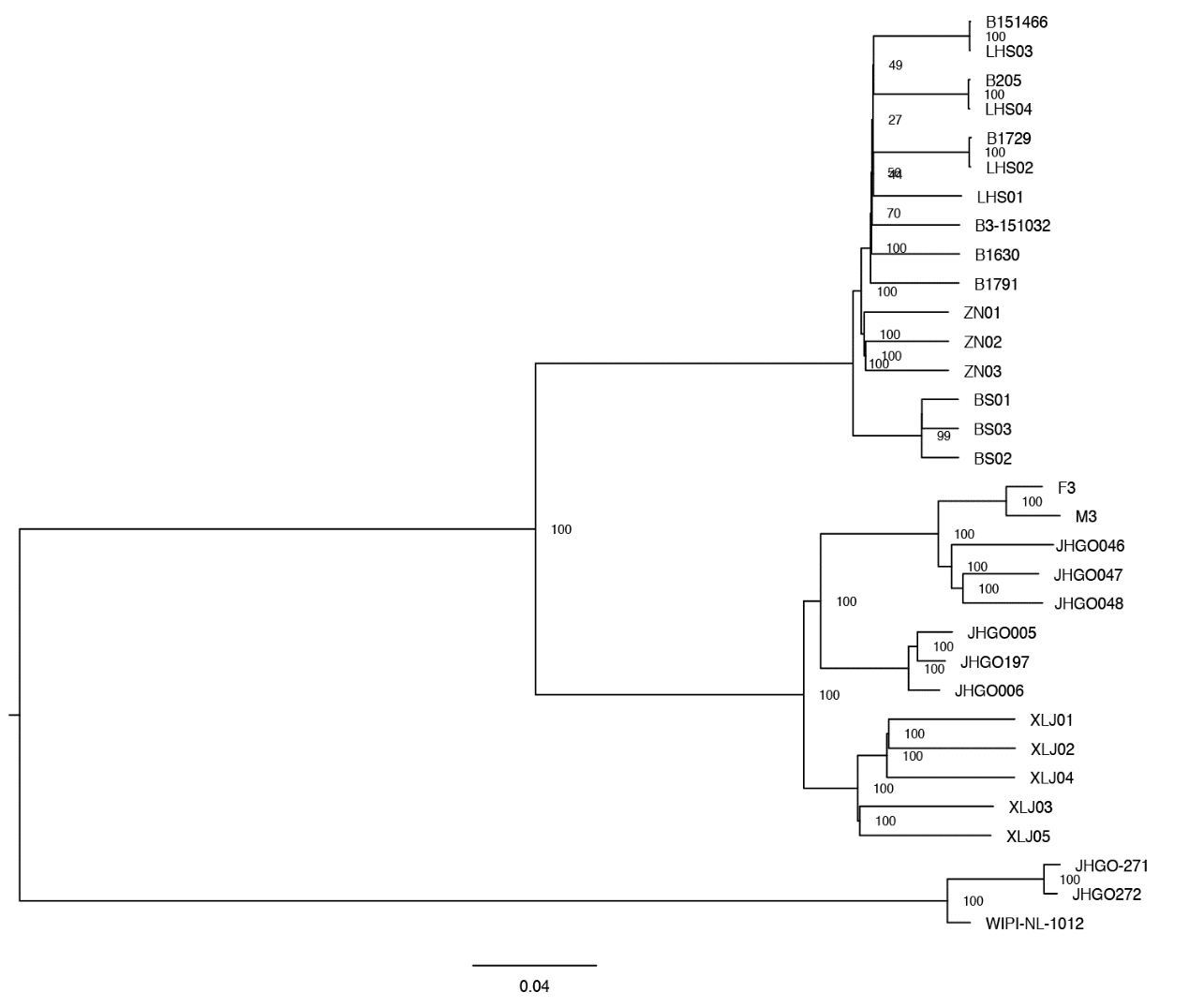


Figure S2. Neighbor-joining tree constructed from Nei’s standard genetic distances of the whole genome sequences of Chinese and Hazel grouse. Numbers at the nodes indicate bootstrap support. Substitution rate is indicated below the figure.
